# Multi-scale Inference of Interaction Rules in Animal Groups Using Bayesian Model Selection

**DOI:** 10.1371/journal.pcbi.1002961

**Published:** 2013-03-21

**Authors:** Richard P. Mann, Andrea Perna, Daniel Strömbom, Roman Garnett, James E. Herbert-Read, David J. T. Sumpter, Ashley J. W. Ward

**Affiliations:** 1 Department of Mathematics, Uppsala University, Uppsala, Sweden; 2 Robotics Institute, Carnegie Mellon University, Pittsburgh, Pennylvania, United States of America; 3 School of Biological Sciences, University of Sydney, Sydney, New South Wales, Australia; Indiana University, United States of America

## Abstract

Inference of interaction rules of animals moving in groups usually relies on an analysis of large scale system behaviour. Models are tuned through repeated simulation until they match the observed behaviour. More recent work has used the fine scale motions of animals to validate and fit the rules of interaction of animals in groups. Here, we use a Bayesian methodology to compare a variety of models to the collective motion of glass prawns (*Paratya australiensis*). We show that these exhibit a stereotypical ‘phase transition’, whereby an increase in density leads to the onset of collective motion in one direction. We fit models to this data, which range from: a mean-field model where all prawns interact globally; to a spatial Markovian model where prawns are self-propelled particles influenced only by the current positions and directions of their neighbours; up to non-Markovian models where prawns have ‘memory’ of previous interactions, integrating their experiences over time when deciding to change behaviour. We show that the mean-field model fits the large scale behaviour of the system, but does not capture the observed locality of interactions. Traditional self-propelled particle models fail to capture the fine scale dynamics of the system. The most sophisticated model, the non-Markovian model, provides a good match to the data at both the fine scale and in terms of reproducing global dynamics, while maintaining a biologically plausible perceptual range. We conclude that prawns’ movements are influenced by not just the current direction of nearby conspecifics, but also those encountered in the recent past. Given the simplicity of prawns as a study system our research suggests that self-propelled particle models of collective motion should, if they are to be realistic at multiple biological scales, include memory of previous interactions and other non-Markovian effects.

## Author Summary

The collective movement of animals in a group is an impressive phenomenon whereby large scale spatio-temporal patterns emerge from simple interactions between individuals. Theoretically, much of our understanding of animal group motion comes from models inspired by statistical physics. In these models, animals are treated as moving (self-propelled) particles that interact with each other according to simple rules. Recently, researchers have shown greater interest in using experimental data to verify which rules are actually implemented by a particular animal species. In our study, we present a rigorous selection between alternative models inspired by the literature for a system of glass prawns. We find that the classic theoretical models do not accurately predict either the fine scale or large scale behaviour of the system. Instead, individual animals appear to be interacting even when completely separated from each other. To resolve this we introduce a new class of models wherein prawns ‘remember‚ their previous interactions, integrating their experiences over time when deciding to change behaviour. These show that the fine scale and large scale behaviour of the prawns is consistent with interactions only between individuals who are close together.

## Introduction

The most striking features of the collective motion of animal groups are the large-scale patterns produced by flocks, schools and other groups. These patterns can extend over scales that exceed the interaction ranges of the individuals within the group [1]–[4]. For most flocking animals, the rules dictating the interactions between individuals, which ultimately generate the behaviour of the whole group, are still not known in any detail. Many ‘self-propelled’ particle models have been proposed for collective motion, each based on a relatively simple set of interaction rules between individuals moving in one, two or three dimensions [2], [5]–[8]. Typically these models implement a simple form of behavioural convergence, such as aligning the focal individual's velocity in the average direction of its neighbours or attraction towards the position of those neighbours. Generally such rules are explicitly kept as simple as possible while remaining realistic, with the aim of explaining as much as possible of collective motion from the simplest constituent parts.

Each of the models in the literature is capable of reproducing key aspects of the large-scale behaviour of one or more biological systems of interest. Together these models help explain what aspects of inter-individual interactions are most important for creating emergent patterns of coherent group motion. With this proliferation of putative interaction rules has come the recognition that some patterns of group behaviour are common to many models, and that different models can have large areas of overlapping behaviour depending on the choice of parameters [4]. Common patterns of collective behaviour are also observed empirically across a diverse range of animal and biological systems. For example, a form of phase transition from disorder to order has been described in species as diverse as fish [9], ants [10], locusts [11], down to cells [12] and bacteria [13]. In all these systems, as density of these species is increased there is a sudden transition from random disordered motion to ordered motion with the group collectively moving in the same direction. These studies indicate that a great deal can be understood about collective behaviour without reduction to the precise rules of interaction.

In many contexts however the rules of interaction are of more interest than the group behaviour they lead to. For example, when comparing the evolution of social behavior across different species, it is important to know if the same rules evolved independently in multiple instances, or whether each species evolved a different solution to the problem of behaving coherently as a group [1]. Recently researchers in the field have become interested in using tracking data from real systems on the fine scale to infer what precise rules of motion each individual uses and how they interact with the other individuals in the group [14]–[19]. This is an important trend in the field of collective motion as we move from a theoretical basis, centred around simulation studies, to a more data-driven approach.

The most frequent approach to inferring these rules has been to find correlations between important measurable aspects of the behaviour of a focal individual and its neighbours. For example, Ballerini *et al.* [14] looked at how a focal individual's neighbours were distributed in space relative to the position of the focal individual itself in a group of starlings. Significant anisotropy in the position of the 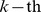 nearest neighbour, averaged over all individuals, was regarded as evidence for an interaction between each bird and that neighbour. More recently Katz *et al.* [18] and Herbert-Read *et al.* [19] investigated how the change in velocity of each individual in groups of fish was correlated to the positions and velocities of the neighbouring fish surrounding the focal individual. This provides evidence not only for the existence of an interaction between neighbours but also estimates the rules that determine that interaction.

In these studies the rules of interaction are presented non-parametrically and cannot be immediately translated into a specific self-propelled particle model. Nor are these models validated in terms of the global schooling patterns produced by the fish. An alternative model-based approach that does fit self-propelled particle and similar models to data is proposed by Eriksson *et al.* [16] and Mann [17]. Under this approach, the recorded fine-scale movements of individuals are used to fit the parameters of, and select between, these models in terms of relative likelihood or quality-of-fit. This approach has the advantage of providing a parametric ‘best-fit’ model and can provide a quantitative estimate the relative probability of alternative hypotheses regarding interactions.

What all previous empirical studies have lacked is a simultaneous verification of a model at both the individual and collective level. Either fine scale individual-level behaviour is observed without explicit fitting of a model [18], [19] or global properties, such as direction switches [11], [20], speed distributions [21], [22] or group decision outcome [23] have been compared between model and data. Verification at multiple scales is the necessary next step now that inference based on fine-scale data is becoming the norm. Just as simulations of large-scale phenomena can appear consistent with observations of group behaviour without closely matching the local rules of interaction, so can fine-scale inferred rules be inconsistent with large-scale phenomena if these rules of inferred from too limited a set of possible models or from correlations between the wrong behavioural measurements. The closest that any study so far has come to finding consistency between scales has been Lukeman *et al.* [15]. In their study the local spatial distribution of neighbouring individuals in a group of scoter ducks was used to propose parametric rules of interaction, with some parameters measured from the fine-scale observables, but with others left free to be fitted using large-scale data. We suggest that if group behaviour emerges from individual interactions, then the form of these interactions should be inferable solely from fine-scale data without additional fitting at the large-scale. An inability to replicate the group behaviour using a selected model demonstrates that the model space has been insufficiently explored. When faced with alternative hypothesised interaction rules, model-based parametric inference provides the best means of quantitatively selecting between them.

In this paper we study the collective motion of small groups of the glass prawn, *Paratya australiensis*. *Paratya australiensis* is an atyid prawn which is widepsread throughout Australia [24]. Although typically found in large feeding aggregations, it does not appear to form social aggregations and has not been reported to exhibit collective behaviour patterns in the wild. We conduct a standard ‘phase transition’ experiment [9], [11], [12], studying how density affects collective alignment of the prawns. We complement this approach by using Bayesian inference to perform model selection based on empirical data at a detailed individual level. We select between models by calculating the probability of the fine scale motions using a Bayesian framework specifically to allow fair comparison between competing models of varying complexity. Comparison of the marginal likelihood, the probability of the data conditioned on the model, integrating over the uncertain parameter values, is a well developed and robust means of model selection that forms the core of the Bayesian methodology [25]–[28] and which has been applied to compare models in the biological sciences, particularly neuroscience [29]. Bayesian methods are also well established in animal behaviour through consideration of optimal decision making in the presence of conflicting information, both environmental [30] and social [31], [32]. In adopting this approach, we reject the dichotomy of model inference based on either fine scale behaviour of the individuals or the motion of the group. Instead we use reproduction of the large scale dynamics through simulation as a necessary but not sufficient condition of the correct model.

## Results

**Figure 1:**
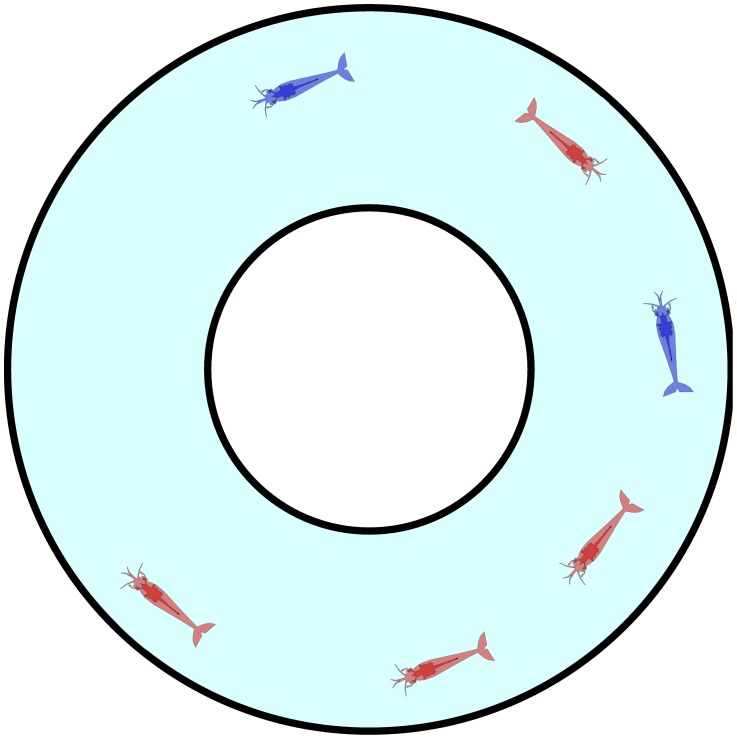
Schematic of the experimental setup. Prawns moving within an annulus of 200 mm external diameter and 70 mm internal diameter. Red coloured prawns indicate a clockwise orientation, blue prawns a counter-clockwise orientation. In this instance the total number of prawns 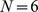, number of clockwise-moving oriented prawns 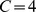, the polarisation 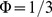, and the excess polarisation 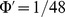.

We study the positions and directions of co-moving prawns in a confined annular arena (See Materials and Methods and Figure 1 and also Figure S1 and Video S1 in the supplementary material). We tracked, using semi-automated software, the position of each prawn through the duration of the experiments. We pre-processed those raw tracking data by using a Hidden Markov Model to classify the movements of each prawn into a binary sequence of clockwise (CW) and anti-clockwise orientation (see Materials and Methods).

**Figure 2:**
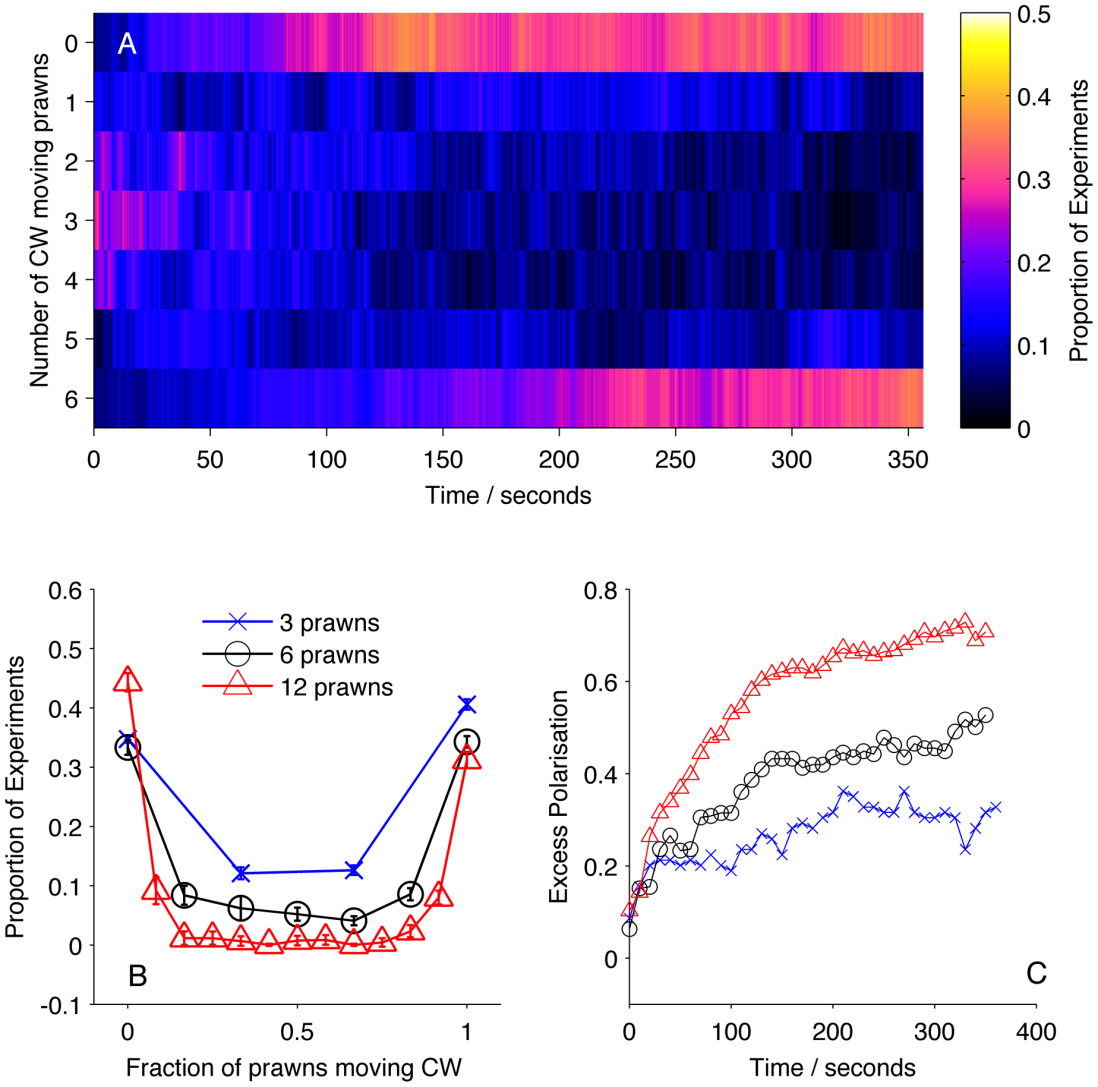
Large-scale behaviour of the experimental system. (A) The proportion of six-prawn experiments (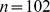) with a given number of CW moving prawns over time. For each point in time we calculated the distribution over all trials of the number of CW prawns. This distribution is then plotted as a heat map. (B) The final distribution of experiments with number of CW moving prawns, for three-, six- and twelve-prawn experiments (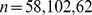 respectively). Error bars represent the mean and standard deviation for each proportion as calculated from the final ten seconds of the experiments. (C) The average polarisation of experiments with three, six and twelve prawns over time, adjusted by the expected polarisation of randomly oriented prawns.

We then calculated the number of prawns travelling CW or anti-CW at each time step of each experiment involving three, six or twelve prawns. From this we calculated the average number of CW and anti-CW prawns at a given time across experiments. Figure 2A shows how the number of CW prawns, 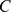, changes over time, taken as a distribution over all trials with six prawns. There is a transition from an initially random configuration, with most trials having 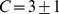, to a final configuration where most experiments have either 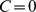 or 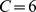. The final stable distribution is further shown in Figure 2B along with the final distribution for three and twelve prawn experiments. Steady state polarisation increases as a function of prawn number. The polarisation, 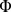 can be defined as
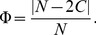
(1)The expected polarisation in randomly oriented groups varies with the number of individuals in the arena, being larger for smaller groups and obeying a binomial distribution. We adjust the measured polarisation by this expectation, 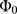, to obtain the excess polarisation, 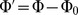. Figure 2C shows this measure of polarisation over time for experiments with three, six and twelve prawns, confirming that the excess polarisation increases over time and is greater for larger groups.

At a group level we see that prawns tend to align over time, producing a polarised stable state, which is higher for larger group sizes. We define the reproduction of these global patterns as the *global consistency condition* of our model. We insist that any realistic model for the prawns' interactions must reproduce this large-scale behaviour.

### Model selection

Next we investigated a series of interaction models as to their ability to reproduce the fine scale interactions of the prawns. We predict the probability, 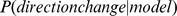, that a focal prawn will change its orientation, given one of a number of potential models. The direction changes are determined by the data from the six-prawn treatment. This treatment provides the best balance between the number of data points, density of direction changes, clear large scale behaviour and tracking accuracy.

Each model specifies the probability that a focal prawn will change its direction in the next time step conditioned on the relative positions and directions of the other individuals in the arena. We use a logistic mapping to ensure probabilities remain between zero and one, so each model uses the relevant variables to determine a latent ‘turning-intensity’, 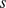, such that,
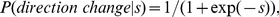
(2)where 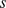 is a function of the relative positions and directions of the other prawns, both now and potentially in the recent past, and the model parameters.

The models are, in increasing degree of complexity, as follows. Firstly to consider models that do not include zones-of-interaction – non-spatial models. We establish a baseline with a *Null* model. This simply posits that direction changes occur at random, at the rate established from the single prawn data, and the prawns do not interact in any way that changes this direction-changing probability. Therefore 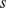 is given simply by a baseline constant, 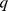, which is determined by the rate of direction changing in single prawns.
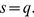
(3)We also consider two models where the interaction is independent of absolute spatial separation. The *Mean Field* (MF) model includes interactions between all prawns regardless of position, such that their relative directions alter the probability of changing direction. Since the number of prawns in the experiment is fixed, the probability for a direction change is influenced by the number of individuals moving in the opposite direction (negative prawns), 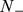. Each negative prawn increases the turning intensity by an amount 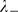,
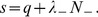
(4)A *Topological* (T) model restricts these interactions to a limited number of nearest-neighbours, 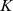, the individuals closest to the focal prawn. The turning intensity is now influenced by the number of negative prawns, 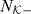 within the set 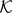 of nearest-neighbours.
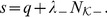
(5)


Secondly we consider a class of *Spatial* models (S1–S4). These models closely resemble the classic one-dimensional self-propelled particle models from the literature [5]. The focal prawn interacts with neighbours within a spatial zone-of-interaction, 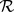. The number and directions of individuals within this interaction zone determine the probability of changing direction. A number of further variations are possible; interactions can be limited to prawns ahead of the focal prawn and/or to prawns travelling in the opposite direction to the focal prawn. We consider four variations, indicated in Table 1. The general form for this model is given by,
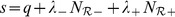
(6)where 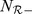 and 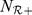 are the number of negative and positive (travelling in the same direction) prawns within the interaction zone, and 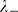 and 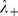 parameterise the influence of each individual on the turning intensity.. Interactions can occur with negative prawns only, 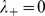, or with both negative and positive oriented prawns, 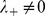. The spatial interaction zone 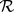 is either a symmetrical area centred on the focal prawn, of width 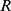 radians around the ring (spatial symmetric models in Table 1), or is only directed 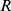 radians ahead of the focal prawn (spatial forward models).

**Table 1:** Model comparison.

Model	Interaction zone	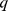	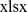 /radians	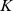	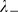	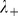	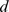	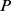 -value	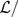 bits
Null	None	−7.5	N/A	N/A	N/A	N/A	N/A	0	−69036
MF	Global	−7.5	N/A	N/A	0.76	N/A	N/A	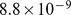	−57976
T	K nearest-neighbours	−7.5	N/A	5	0.77	N/A	N/A	0.077	−58114
S1	Spatial, symmetric	−7.5	0.20 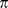	N/A	1.35	N/A	N/A	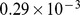	−60035
S2	Spatial, forward	−7.5	0.16 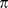	N/A	1.37	N/A	N/A	0	−59102
S3	Spatial, symmetric	−7.5	0.20 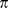	N/A	1.72	0.23	N/A	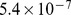	−62297
S4	Spatial, forward	−7.5	0.19 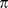	N/A	1.69	0.52	N/A	0	−62004
D1	Spatial, symmetric	−7.5	0.18 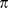	N/A	1.08	N/A	0.87	0.097	−58094
D2	Spatial, forward	−7.5	0.19 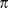	N/A	0.75	N/A	0.94	0.15	−58499
D3	Spatial, symmetric	−7.5	0.19 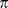	N/A	0.99	−3.59	0.92	0.17	−57963
D4	Spatial, forward	−7.5	0.19 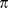	N/A	1.08	0.32	0.92	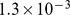	−58512

**Figure 3:**
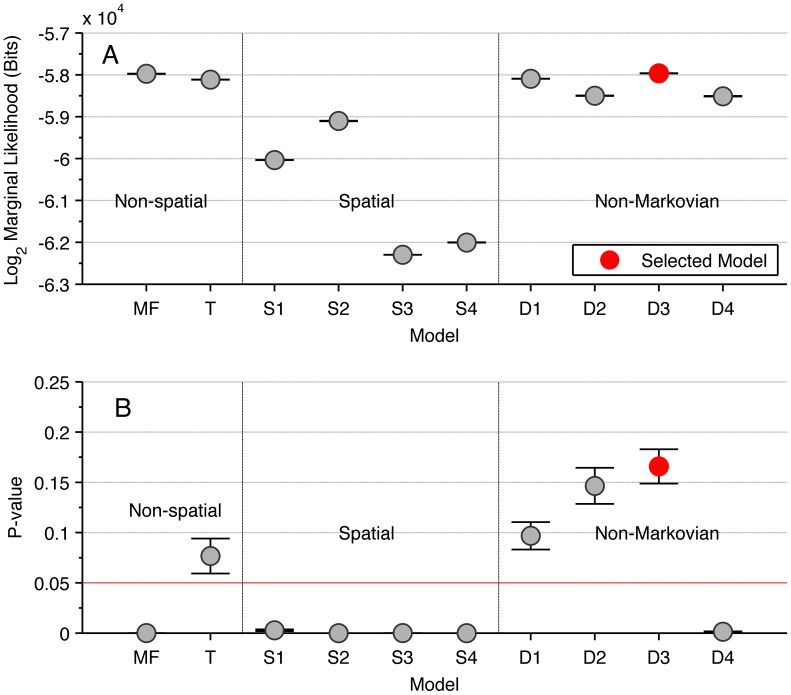
The performance of different models on the fine and large scale. (A) The marginal-likelihood of each model (excepting the null model), calculated from the fine scale dynamics. Each marginal-likelihood is estimated by annealed importance sampling [47]. (B) The p-value associated with the quality-of-fit test between the distributions of model simulation and experimental outcomes (proportion of prawns travelling clockwise at the conclusion of the trial). Each test is performed on 10 independent sets of 100 simulations. On both measures model D3 is the best-performing model, indicating that the focal prawn interacts with all individuals within a short-range symmetric interaction zone, with a ‘memory’ of these interactions that has a persistent influence on the probability of changing direction. Note that the null model has a lower marginal-likelihood and p-value than all other models and is not shown to preserve the scale of the plot.

The interaction zone structure of each model, along with the (maximum *a posteri*) inferred values of model parameters, the 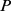-value indicating quality-of-fit between experimental results and model simulations and the log marginal likelihood (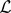) of the model calculated from the fine scale dynamics (as shown in Figure 3. 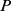-values reported as zero are smaller than numerical precision, *i.e* 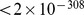). N/A indicates that the model does not include the indicated parameter. The interaction zone indicates whether prawns interact with others in a spatial zone around themselves, which may be oriented either forwards or symmetrically around their centre, or with their nearest-neighbours or globally with all other individuals. Reported parameters are: 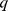, the baseline direction-change intensity; 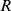, the interaction radius for spatial models; 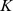, the number of interacting nearest-neighbours for topological models; 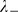 and 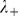, the strength of interaction with negative and positive prawns respectively; and 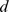, the decay factor determining how long interaction effects persist.

Visual inspection of the movements of the prawns suggests that interactions often follow a particular pattern. Two prawns, travelling in the opposite directions, collide. After the prawns have passed each other one of the prawns may subsequently decide to change direction. Self-propelled particle and other models of collective motion do not capture this type interaction. Such interactions are non-Markovian, *i.e.* the change in direction is not just the result of the environment *now*, but of the past environment as well. We proposed a third class of models (D1–D4), simple *non-Markovian* extensions of the basic spatial models, where each prawn would ‘remember’ the other individuals it encountered, with those memories fading at an unknown rate after the interaction was complete. As such the prawn would integrate those interactions over time, building up experiences which would alter its chance of changing direction. Mathematically this means that the turning intensity is now auto-regressive, depending on its own value at the previous time step as well as the current positions and directions of the neighbouring individuals. We introduce a decay parameter, 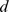, which determines how quickly the turning intensity returns to normal after an interaction with a neighbour has occurred. The same variations of interaction are allowed as for the spatial models, giving a general form for the non-Markovian turning intensity as,
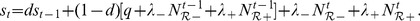
(7)where 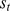 now indicates the turning intensity at time 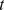, which depends on the value of the turning intensity at the previous time step, 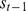. The number of prawns still in the interaction zone from time 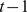 is indicated by 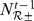, while the number of new arrivals in the interaction zone is given by 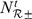. Hence raised (or lowered) turning intensities persist over time, with a duration controlled by the value of 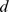. After the focal prawn changes direction the turning intensity is reset to the baseline, 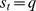, at the next time step.

Table 1 specifies the interaction zone structure for each of eleven alternative models, grouped according to the description given above. For each model we calculate the marginal likelihood of the data, conditioned on the interaction model (see Materials and Methods). The marginal likelihood is the appropriate measure for performing model selection, especially between models of varying complexity. More complex models, by which we mean models with a larger number of free parameters, are penalised relative to simpler models when integrating over the parameter space, since less probability can be assigned to any particular parameter value *a priori*. The marginal likelihood indicates how likely a particular model is, rather than a model and an chosen optimal parameter value (see, for example, Mackay [33] Chapter 28 and other standard texts for discussions on this topic). The marginal likelihoods of each model are shown in Figure 3A.

We also measure the consistency between the large scale results of our experiments and the results predicted by simulation of each model, using the parameter values in Table 1. We set a consistency condition that any model that accurately approximates the true interactions must fulfil. We measure the large scale quality-of-fit between the model simulations and the experiments using the Kullback-Leibler divergence [34] between the distribution of simulated and experimental outcomes and performing a G-test for quality-of-fit [35] (see Materials and Methods). The p-value associated with this quality-of-fit for each model is shown in Figure 3B, showing which models are deemed to be consistent with experiments (those with 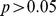). Large scale results from the simulation of each model are shown individually in Figures S2, S3, S4, S5, S6, S7, S8, S9, S10, S11, S12 in the supplementary materials.

**Figure 4:**
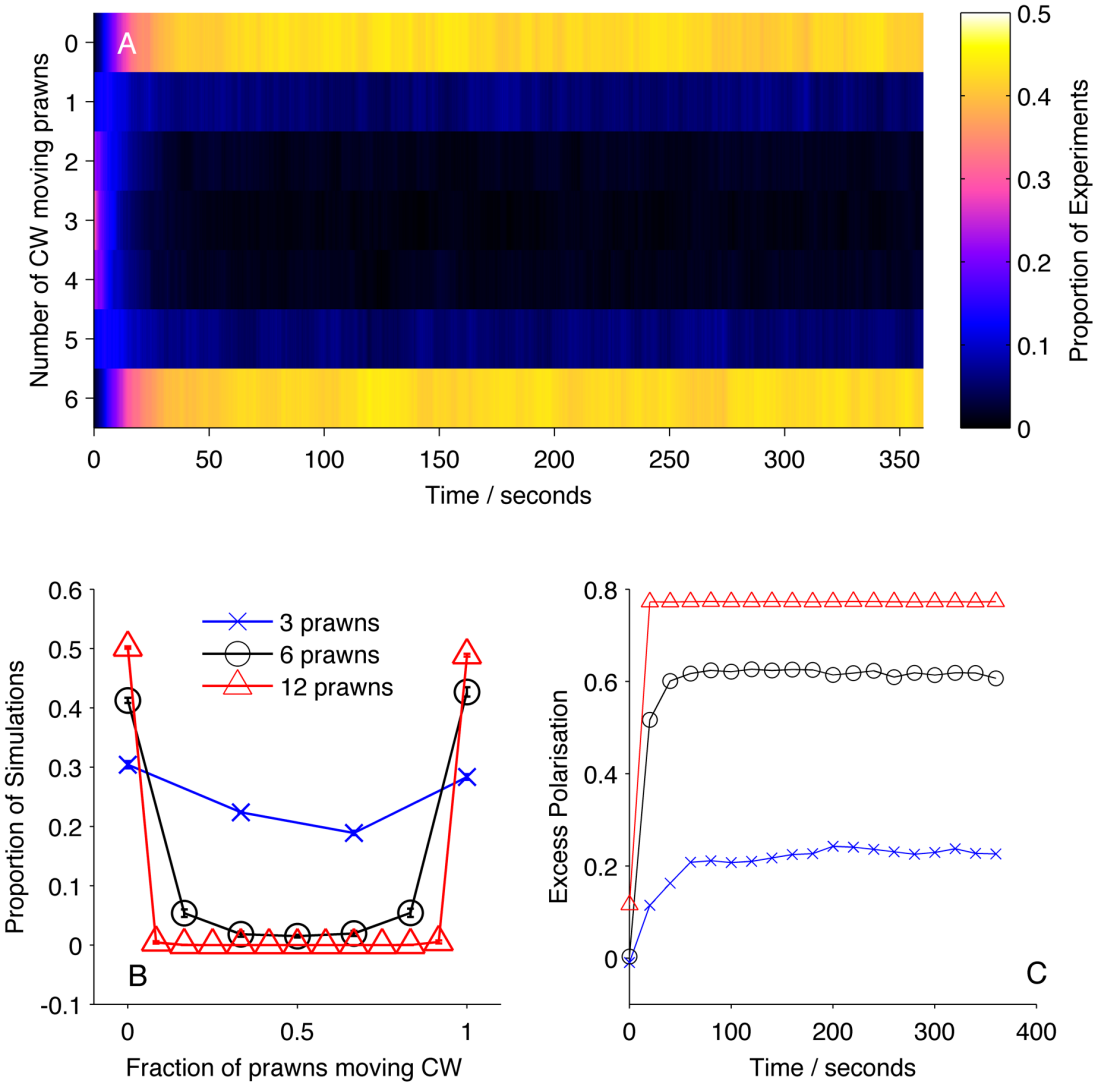
Simulation results for mean-field model MF. (A) Proportion of six-prawn simulations (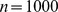) of mean-field model MF with a given number of prawns moving CW over time. (B) Final distribution of simulations by number of CW moving prawns for simulations with three, six and twelve prawns. Error bars represent the mean and standard deviation for each proportion as calculated from the final ten seconds of the simulations. (C) The average polarisation over time, adjusted by the expected polarisation of randomly oriented prawns, for simulations of three, six and twelve prawns.

The Null model, in which prawns do not interact, performs significantly worse than the mean-field model. Figure 4 shows that the mean-field reproduces both the global alignment of the prawn groups, with an increase in polarisation with time and group size. These results show that the prawns interactions involve matching their directions to that of others, producing alignment.

**Figure 5:**
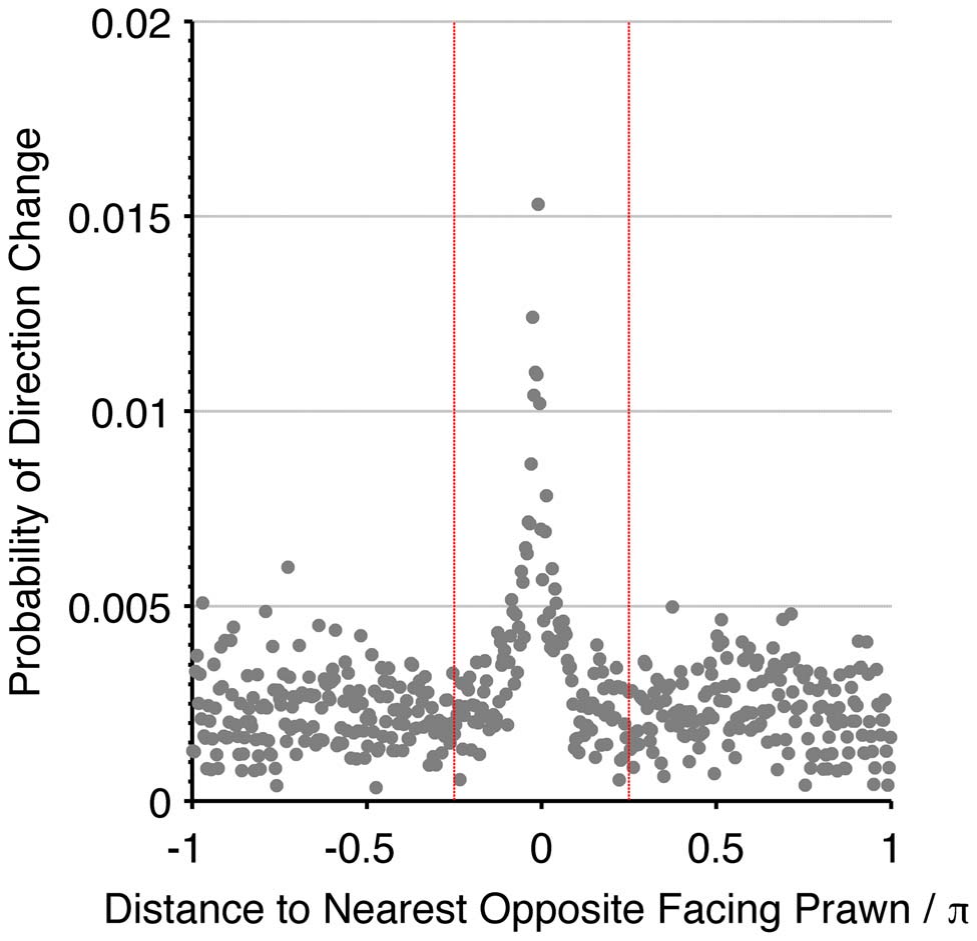
Evidence for short-range interactions. The empirical frequency of direction changing as a function of the distance to the nearest opposite facing prawn (grey markers). The empirical data clearly shows the spatially localised interaction with a central peak. The red dashed lines indicate a region of 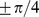 radians, which confines the interaction peak and informs our prior probability distribution on the possible interaction range.

Are local spatial interactions important in reproducing observed direction changes? We note first that a topological interaction zone, where the focal prawn interacts with its 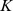 nearest neighbours, has a marginal likelihood slightly lower than the mean field model. The topological model is ‘punished’ for having more parameters than the mean-field model, since the most probable value of the topological interaction range encompasses all neighbours. However, interactions between prawns *are* local. Figure 5 shows how the probability of changing direction depends on the position of the nearest opposite facing neighbour. An opposite facing neighbour within approximately 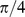 radians of a focal prawn strongly increases the chance that the focal prawn will change direction.

This observation suggests that a local interaction spatial model should outperform the mean field model, and we can use the approximate observed range of interaction (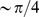 radians) to inform our prior probability on the interaction zone for models that include one. However, Figure 3A shows that with this limit on the interaction zone, the spatial models (S1–S4) all have a marginal likelihood lower than the mean field model. Simulating these models with most-probable parameters inferred from our analysis of the data (see Table 1) shows that these fit poorly on the large scale too, having a relatively large divergence between the simulated outcomes and the observed large scale alignment patterns and are therefore showing significant differences in the quality-of-fit test (Figure 3B). Both Figure 5 and our biological reasoning insist that locality must be maintained in interactions between individual animals. Therefore the poor performance of these spatial models indicates that they are an incomplete description of the true behaviour of the prawns.

**Figure 6:**
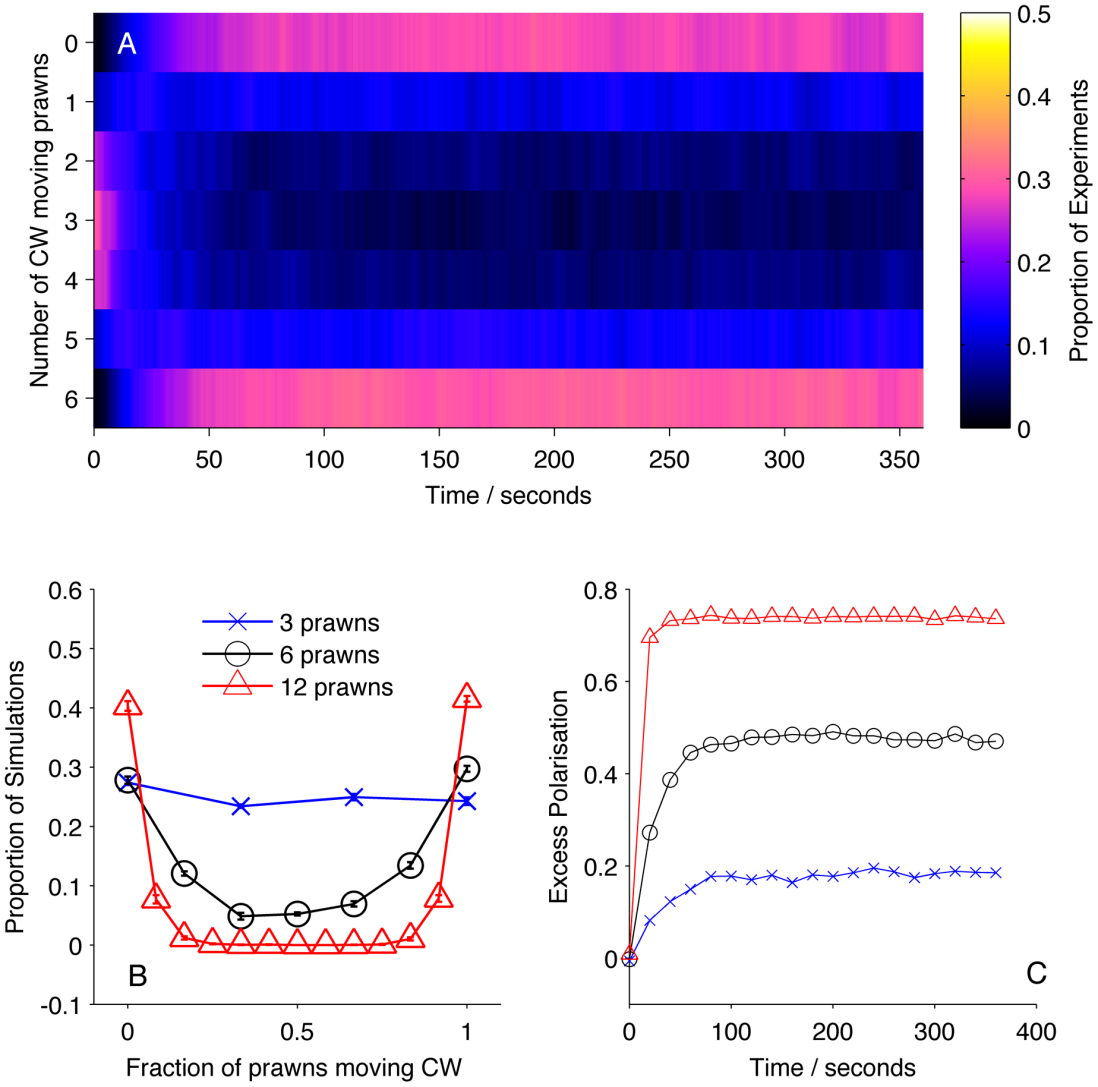
Simulation results for non-Markovian model D3. (A) Proportion of six-prawn simulations (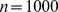) of non-Markovian model D3 with a given number of prawns moving CW over time, showing a bifurcation to either a CW or an anti-CW polarised state, with most simulations concluding with six prawns travelling in the same direction. (B) Final distribution of simulations by number of CW moving prawns for simulations with three, six and twelve prawns. Error bars represent the mean and standard deviation for each proportion as calculated from the final ten seconds of the simulations. (C) The average polarisation over time, adjusted by the expected polarisation of randomly oriented prawns, for simulations of three, six and twelve prawns.

The models incorporating a non-Markovian delayed response together with a spatial interaction zone (models D1–D4) all outperformed the most probable Markovian spatial model on both the fine and large scales (Figure 3). Model D3 is the best performing model on both scales, and is the only model with a greater marginal likelihood than the Mean Field model. This then is the best model we can infer from our selection of possibilities. Figure 6 shows that simulations of model D3 produce collective alignment of the prawns and consistently stronger and faster alignment for larger group sizes, fulfilling our large-scale consistency requirement for a realistic model. The inferred value of the memory parameter associated with this model (see Table 1) puts the half-life of these memories at approximately one second. Combined with the average angular speed of the prawns (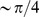 radians/s) this means that prawns can be separated by a full half of the arena while still exerting a considerable influence on each other's behaviour. This potentially explains the strong performance of the mean field model in explaining the fine scale interactions between individuals.

## Discussion

A number of physical [36]–[38], technological [39] and biological systems, including animals [9]–[11], [40], tissue cells [12], microorganisms [13], [41] are known to increase their collective order with density. Glass prawns are one additional example of such a system, which is particularly interesting since they are not known as gregarious or social species. By confining the prawns to a ring we facilitated their interactions and in doing so generated collective motion. This adds further support to the idea that collective motion is a universal phenomenon independent of the underlying interaction rules [4], [11], [42]. While we do not expect that prawns often find themselves confined in rings in a natural setting, they and other non-social animals do aggregate in response to environmental features such as food and shelter. Such environmental aggregations can, above a certain density, result in an apparently ‘social’ collective motion.

The true value of this study, however, is found not in the addition of one more species to this growing list, but in demonstrating a rigorous methodology for selecting an optimal and multi-scale consistent model for the interactions between individuals in a group. We have used a combination of techniques to identify the optimal model for our experiments: Bayesian model selection, validation against global properties and consistency with biological reasoning. We applied Bayesian model selection to identify the model that best predicts the fine-scale interactions between prawns. This approach allows us to perform model selection in the presence of many competing hypotheses of varying complexity, while avoiding over fitting [17]. This indicated the selection of a non-Markovian model with a persistent ‘memory’ effect. We find that interactions are governed by a perceptual range which is symmetric about the focal individual which is somewhat greater than the average body length of the prawns (approximately 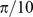 radians).

Reproduction of the large-scale dynamics is frequently used to validate mathematical models of biological systems, but presents only a necessary and not a sufficient condition for model validation. Indeed, all of the models we have assessed in this work can, with the appropriate parameters, generate aligned motion consistent with experiment. The fact that our mean-field model reproduces global dynamics, but fails at a fine-scale level is not particularly surprising. Mean-field models are not designed to reproduce spatially local dynamics [1]. More illuminating, however, is the failure of Markovian spatial models to reproduce the fine-scale dynamics when the locality of interactions between individuals is imposed. Models S1, S2, S3, S4 are variants of the standard one dimensional Vicsek self-propelled particle model [43], which has previously been validated against the global alignment patterns of marching locusts [11]. For the prawns these models perform poorly on both capturing the fine scale dynamics of interactions and in reproducing the large scale alignment patterns seen in the data. This inconsistency allowed us to reject standard self-propelled particle models as a good model of the data.

To identify a better model we first visually inspected the interactions between the prawns. These observations suggested a ‘memory effect’, whereby a prawn would remain influenced by individuals beyond the moment of interaction. The resulting models are able reproduce the fine scale and large scale dynamics of the prawns, while also maintaining the biologically-intuitive locality of interactions between individuals. More generally, we would expect other examples of animal motion to be non-Markovian, with individuals taking time to react to others, to complete their own actions and also potentially reacting through memory of past situations. In this context, it is important to consider the limitations of recent studies identifying rules of interaction of fish [18], [19]. These studies concentrated on quantifying local interactions, but do not try to reproduce global properties. It may be that non-Markovian and other effects are needed to produce these properties.

In what circumstances can we expect non-Markovian effects to play an important role in collective behaviour? Inference based on a Markovian model must account for behavioural changes of a focal individual in terms of their current environment. As such the crucial factor is how much the local environment changes between when the animal receives information and when it responds. Large changes in the local environment can be caused by long response times or by rapid movements of other animals relative to the focal individual. Where behavioural changes are strongly discontinuous, such as the binary one-dimensional movement in this study, non-Markovian effects may become especially important. This is because the focal individual may have to execute a number of small changes (such as stopping and turning through a several small angles) in order to register as having changed its direction of motion. Over the course of making many adjustments the environment can change dramatically from the moment that the change was initiated.

We have compared the models on the large scale by evaluating the quality-of-fit between the distribution of large scale outcomes predicted by model simulations with that seen in experiments. The model we select from the fine scale analysis is also evaluated as the best on this large scale analysis, and produces simulation results that are qualitatively consistent with experiment (see Figure 6). Because the same model is selected from both analyses we have not been forced to weight the relative importance of each. In future it may be necessary to decide on an appropriate weighting of these different criteria where they disagree on the optimal model. The research presented here provides a first step towards the use of multi-scale inference in the study of collective animal behaviour and in other multi-level complex systems.

## Materials and Methods

Glass prawns (*Paratya australiensis*) were collected from Manly Dam, Sydney, Australia and transported back to aquaria facilities at the University of Sydney. They were held in 20 glass aquaria and fed green algae and fish food ad libitum. Prawns were housed for at least 2 days prior to experimentation. An annulus arena (200 mm external diameter, 70 mm internal diameter) was constructed from white plastic and filled to a depth of 25 mm with freshwater. The arena was visually isolated inside an opaque white box and filmed from above using a G10 Canon digital camera at a frame rate of 15 Hz. Data was subsequently down-sampled to 7.5 Hz by removing every second frame for computational efficiency. For each trial, we haphazardly selected one, three, six or twelve prawns and placed them in the arena. We filmed each trial for six minutes, after which we removed the prawns, emptied, and then refilled the arena with freshwater. Prawns were only used once on each day of trials. A schematic of this setup is shown in Figure 1.

### Hidden Markov Model

The frame-by-frame movements of the prawns are imperfect representations of the true orientation, since a prawn will often stop or even drift slightly backwards without physically turning around. A Hidden Markov Model (HMM) allows the underlying orientation of the prawns to be discovered from the noisy frame-by-frame movements by demanding a higher degree of ‘evidence’ for a direction change, in essence only identifying direction changes when the prawn makes a sustained movement in the new direction. This gives a better estimate of the true orientation than given by the instantaneous velocity alone.

**Figure 7:**
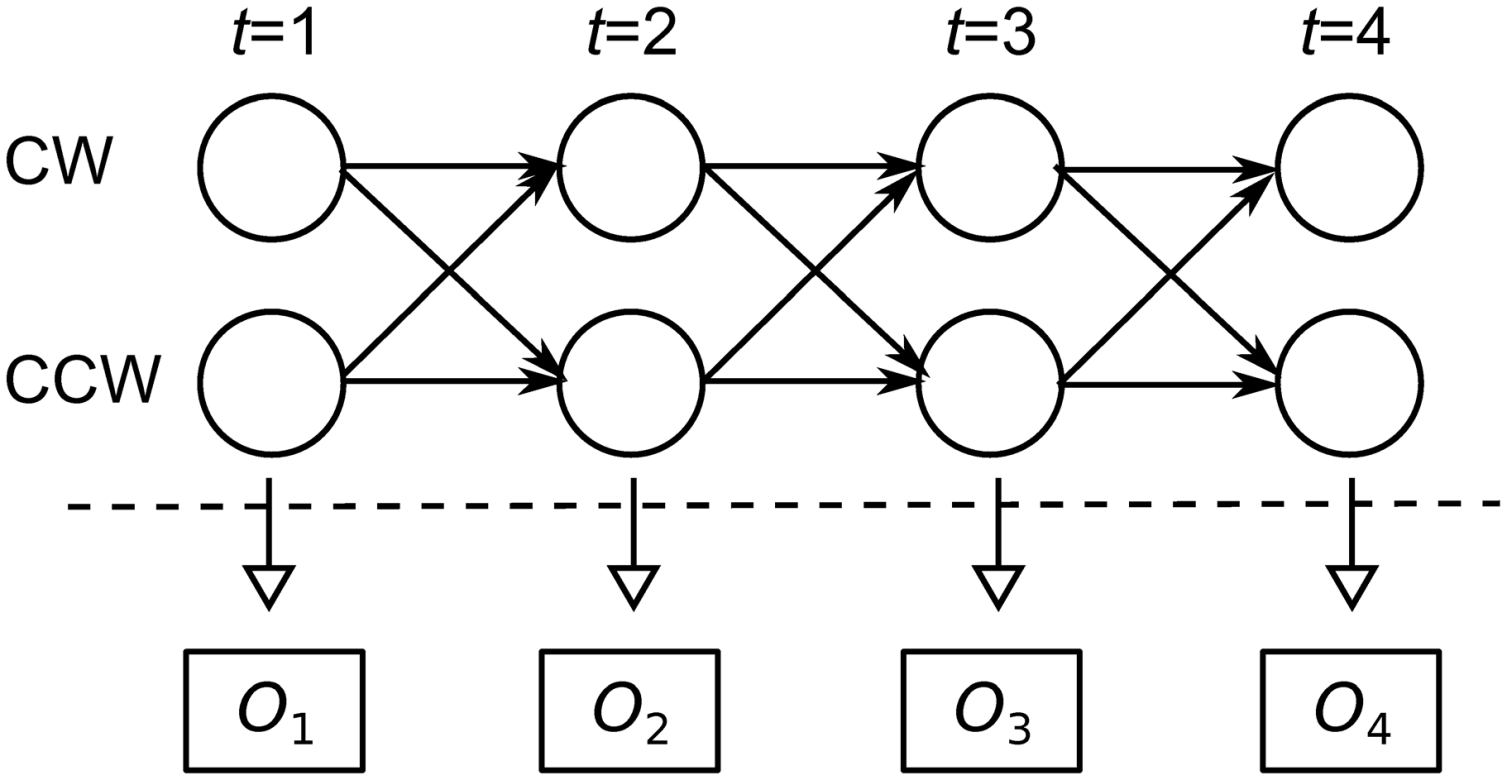
Graphical description of a two-state Hidden Markov Model. At any point in time the prawn is in a state of either CW or anti-CW orientation. The precise state is hidden but we make observations 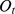, the actual frame-by-frame movements of the prawn, which give information about the relative probabilities of the two states. We assume a fixed probability of transition between the states which is inferred from the data and allows for the persistence of orientation over time.

We constructed a two-state HMM [44] for the observed changes in position of the prawn, as shown in Figure 7. The two states represent clockwise (CW) or anti-clockwise (anti-CW) orientation. In a CW oriented state it is assumed that the prawn will normally move in CW direction over the course of one frame, but because the prawns movements are noisy it may move in the reverse direction over short time periods while remaining oriented CW. We model the distribution of these movements as a Gaussian distribution. We further assume a symmetrical model, such that the distribution of movements in the CW state is anti-symmetric to the distribution of movements in the anti-CW state. Thus a movement of zero is equally probable in either state. We use the Baum-Welch algorithm [44], [45] to learn the transition probability and the mean and standard deviation of the Gaussian observation probability distribution, using data from single-prawn experiments. We then apply this learnt model to identify the most probable state sequence for each of the prawns in the three-, six- and twelve-prawn experiments, using the Viterbi algorithm [44], [46]. We further reduce the number of artifactual detected direction changes by removing any instances where a prawn changes direction twice within one second, since inspection suggests these events are caused by tracking errors.

### Calculation of marginal likelihoods for fine scale comparison

A given model, 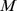 describes the probability of a change of direction for the focal prawn at time 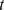, conditioned on the current, and potentially past, positions of the other prawns, 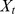 and 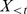 and the parameters of the model 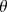. The likelihood for a given parameter set of the model is the probability of the data, 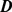, conditioned on the parameters and the model and is the product over both time steps and focal prawns of the probability for the observed outcome - either a change of direction or no change. Let 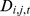 equal one when prawn 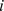 in experiment 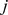 changes direction at time 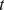, and is zero otherwise, then,
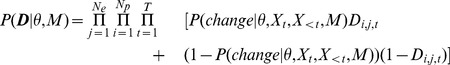
(8)where 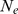 and 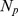 indicate the number of experiments and the number of prawns in each experiment respectively. The marginal likelihood of the model is given by integration over the space, 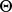, of unknown parameters,
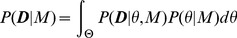
(9)The prior distribution of the parameters, 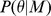 is chosen to represent the available knowledge about the parameters and is split into independent parts. We use the empirical observations in Figure 5 to inform the prior distribution on the interaction range and possible interaction strengths. The prior distribution over the number of interacting neighbours in the topological model is set to the entire possible range for the analysed six-prawn experiments, and the prior distribution for the memory factor is naturally between 0 (no memory) and 1 (permanent memory). The prior for the same parameter over different models is the same to allow fair comparison.
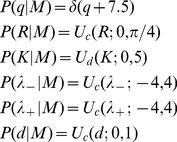
(10)where 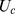 indicates a continuous uniform distribution, 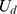 indicates a discrete uniform distribution and 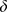 is the Dirac delta function. Numerical integration over the appropriate parameters was performed using annealed importance sampling [47], with 1000 parameter samples.

### Inference of most probable parameter values

We select the most probable parameter values, 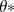 for each model as those which maximise the posterior probability distribution,
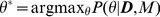
(11)where the posterior probability distribution is given in terms of the likelihood, prior distribution and model evidence defined above
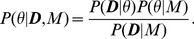
(12)In practice we evaluate the posterior probability for each parameter sample generated within the annealed importance sampling algorithm [47] and select the most probable for each model.

### Model simulation

Given the most probable parameter values (maximum *a posteri*) for a given model inferred from the fine scale data via equation 12, simulations of that model can be performed to assess the likely large scale results of the interactions the model encodes. To perform these simulations we treat individual prawns as particles moving on a circular ring. Each particle is initially set to have either CW or CCW motion at random. At each time step each particle, taken in a random order, moves around the ring in its direction of motion, moving a distance sampled from a distribution matched to the mean and variance of the experimentally observed motions (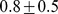 radians/s). After this motion, the distance between all the particles is calculated, and for each particle a decision is made whether to change the direction of motion, based on the rules encoded by the model being simulated. The time step used is 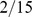 s, which is matched to the time spacing in the analysed data.

### Calculation of Kullback-Leibler divergences for large scale comparison

It is observed in model simulations that the rate at which the group aligns is highly dependent on the speed of individuals, which we have not attempted to model accurately. However, the final state after 360 seconds of simulation (the length of the experiments) is not sensitive to this factor. Therefore we evaluate the quality-of-fit between the model and experimental data by examine the distribution of final states in the experiments and simulations – that is, how many individuals are travelling clockwise when the experiment or simulation ends. We average this over the final 10 seconds of the experiment or simulation to increase the accuracy of this judgement. The quality-of-fit for the model is given by the Kullback-Leibler (KL) divergence [34], 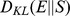 from the experimental distribution of outcomes, 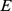 to the simulated distribution, 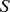. This is a canonical measure of how well one distribution (the simulated outcomes) approximates another (the experimental outcomes). If 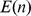 is the proportion of experiments where 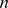 prawns are travelling clockwise, and similarly 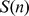 the proportion of simulations where 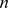 particles are travelling clockwise, then the divergence is given by
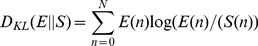
(13)where 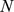 is the total number of prawns in the experiment or simulation. We calculate this divergence between experiment and simulation for scenarios with 3, 6 and 12 prawns to check for consistency over varying group size. The statistical significance of these divergences can be calculated using the G-statistic, 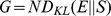, where 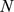 is the number of experiments, and the KL divergence is evaluated using the natural logarithm. The null hypothesis that the experimental results come from the simulated distribution implies a 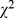-distribution for the G-statistic [35].

### Note

This article is a revised version of a paper of the same title [48] that was previously published in PLOS Computational Biology and was subsequently retracted when a computational error was discovered.

## Supporting Information

Figure S1
**Image of the experimental setup.** Prawns moving within an annulus of 200 mm external diameter and 70 mm internal diameter. In this instance the total number of prawns 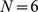, number of clockwise-moving oriented prawns 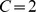, the polarisation 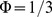, and the excess polarisation 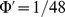

(TIFF)

Figure S2
**Simulation results for model 0.** (A) Proportion of six-prawn simulations (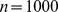) with a given number of prawns moving CW over time. (B) Final distribution of simulations by number of CW moving prawns for simulations with three, six and twelve prawns. Error bars represent the mean and standard deviation for each proportion as calculated from the final ten seconds of the simulations. (C) The average polarisation over time, adjusted by the expected polarisation of randomly oriented prawns, for simulations of three, six and twelve prawns.(TIFF)

Figure S3
**Simulation results for model MF.** (A) Proportion of six-prawn simulations (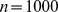) with a given number of prawns moving CW over time. (B) Final distribution of simulations by number of CW moving prawns for simulations with three, six and twelve prawns. Error bars represent the mean and standard deviation for each proportion as calculated from the final ten seconds of the simulations. (C) The average polarisation over time, adjusted by the expected polarisation of randomly oriented prawns, for simulations of three, six and twelve prawns.(TIFF)

Figure S4
**Simulation results for model Topo.** (A) Proportion of six-prawn simulations (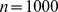) with a given number of prawns moving CW over time. (B) Final distribution of simulations by number of CW moving prawns for simulations with three, six and twelve prawns. Error bars represent the mean and standard deviation for each proportion as calculated from the final ten seconds of the simulations. (C) The average polarisation over time, adjusted by the expected polarisation of randomly oriented prawns, for simulations of three, six and twelve prawns.(TIFF)

Figure S5
**Simulation results for model S1.** (A) Proportion of six-prawn simulations (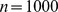) with a given number of prawns moving CW over time. (B) Final distribution of simulations by number of CW moving prawns for simulations with three, six and twelve prawns. Error bars represent the mean and standard deviation for each proportion as calculated from the final ten seconds of the simulations. (C) The average polarisation over time, adjusted by the expected polarisation of randomly oriented prawns, for simulations of three, six and twelve prawns.(TIFF)

Figure S6
**Simulation results for model S2.** (A) Proportion of six-prawn simulations (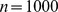) with a given number of prawns moving CW over time. (B) Final distribution of simulations by number of CW moving prawns for simulations with three, six and twelve prawns. Error bars represent the mean and standard deviation for each proportion as calculated from the final ten seconds of the simulations. (C) The average polarisation over time, adjusted by the expected polarisation of randomly oriented prawns, for simulations of three, six and twelve prawns.(TIFF)

Figure S7
**Simulation results for model S3.** (A) Proportion of six-prawn simulations (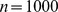) with a given number of prawns moving CW over time. (B) Final distribution of simulations by number of CW moving prawns for simulations with three, six and twelve prawns. Error bars represent the mean and standard deviation for each proportion as calculated from the final ten seconds of the simulations. (C) The average polarisation over time, adjusted by the expected polarisation of randomly oriented prawns, for simulations of three, six and twelve prawns.(TIFF)

Figure S8
**Simulation results for model S4.** (A) Proportion of six-prawn simulations (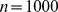) with a given number of prawns moving CW over time. (B) Final distribution of simulations by number of CW moving prawns for simulations with three, six and twelve prawns. Error bars represent the mean and standard deviation for each proportion as calculated from the final ten seconds of the simulations. (C) The average polarisation over time, adjusted by the expected polarisation of randomly oriented prawns, for simulations of three, six and twelve prawns.(TIFF)

Figure S9
**Simulation results for model D1.** (A) Proportion of six-prawn simulations (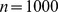) with a given number of prawns moving CW over time. (B) Final distribution of simulations by number of CW moving prawns for simulations with three, six and twelve prawns. Error bars represent the mean and standard deviation for each proportion as calculated from the final ten seconds of the simulations. (C) The average polarisation over time, adjusted by the expected polarisation of randomly oriented prawns, for simulations of three, six and twelve prawns.(TIFF)

Figure S10
**Simulation results for model D2.** (A) Proportion of six-prawn simulations (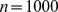) with a given number of prawns moving CW over time. (B) Final distribution of simulations by number of CW moving prawns for simulations with three, six and twelve prawns. Error bars represent the mean and standard deviation for each proportion as calculated from the final ten seconds of the simulations. (C) The average polarisation over time, adjusted by the expected polarisation of randomly oriented prawns, for simulations of three, six and twelve prawns.(TIFF)

Figure S11
**Simulation results for model D3.** (A) Proportion of six-prawn simulations (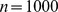) with a given number of prawns moving CW over time. (B) Final distribution of simulations by number of CW moving prawns for simulations with three, six and twelve prawns. Error bars represent the mean and standard deviation for each proportion as calculated from the final ten seconds of the simulations. (C) The average polarisation over time, adjusted by the expected polarisation of randomly oriented prawns, for simulations of three, six and twelve prawns.(TIFF)

Figure S12
**Simulation results for model D4.** (A) Proportion of six-prawn simulations (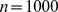) with a given number of prawns moving CW over time. (B) Final distribution of simulations by number of CW moving prawns for simulations with three, six and twelve prawns. Error bars represent the mean and standard deviation for each proportion as calculated from the final ten seconds of the simulations. (C) The average polarisation over time, adjusted by the expected polarisation of randomly oriented prawns, for simulations of three, six and twelve prawns.(TIFF)

Text S1 **A summary of provided supplementary figures and videos.**
(PDF)

Video S1 **Video of a single experiment with six prawns.**
(M4V)
