# Supplementary figures and images for: Multi-scale Inference of Interaction Rules in Animal Groups Using Bayesian Model Selection
Source: PLoS Comput Biol. 2013 Mar 21;9(3):e1002961. doi: 10.1371/journal.pcbi.1002961 (PMC3605063; doi:10.1371/journal.pcbi.1002961)

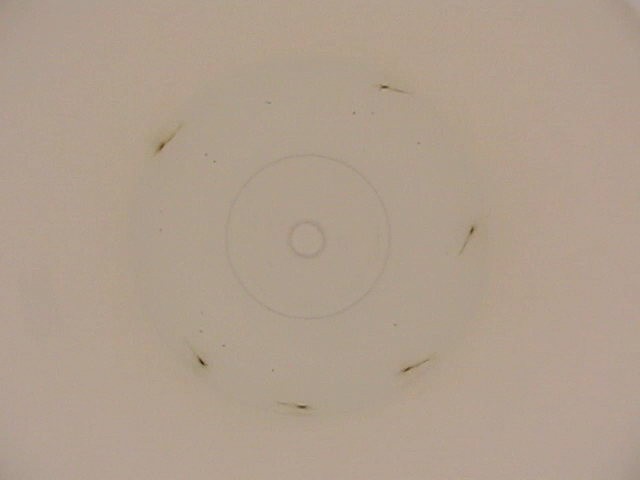

Supplement: Figure S1 — Image of the experimental setup. Prawns moving within an annulus of 200 mm external diameter and 70 mm internal diameter. In this instance the total number of prawns , number of clockwise-moving oriented prawns , the polarisation , and the excess polarisation (TIFF) [file pcbi.1002961.s001.tif]
