# Supplementary material for: Multi-scale Inference of Interaction Rules in Animal Groups Using Bayesian Model Selection
Source: PLoS Comput Biol. 2013 Mar 21;9(3):e1002961. doi: 10.1371/journal.pcbi.1002961 (PMC3605063; doi:10.1371/journal.pcbi.1002961)
Supplement: Text S1 — A summary of provided supplementary figures and videos. (PDF) [file pcbi.1002961.s013.pdf]

Text S1:

The experimental setup

Figure S1 shows a still image of the experimental setup from a typical experiment with six prawns. Video S1 shows a video of the same experiment.

Simulation results for all tested models

Figures S2-S12 show the results of simulating each of the models tested in this paper, using the maximum *a posteri* parameters inferred from the data shown in Table 1. The quantitative quality-of-fit between these and the experimental data is shown in Figure 3.
